# Long-term dynamics of the abundance of earthworms and enchytraeids (Annelida, Clitellata: Lumbricidae, Enchytraeidae) in forests of the Central Urals, Russia

**DOI:** 10.3897/BDJ.9.e75466

**Published:** 2021-11-26

**Authors:** Evgenii Vorobeichik, Alexey Nesterkov, Elena Golovanova, Dina Nesterkova, Alexander Ermakov, Maxim Grebennikov

**Affiliations:** 1 Institute of plant and animal ecology, UB RAS, Yekaterinburg, Russia Institute of plant and animal ecology, UB RAS Yekaterinburg Russia; 2 Omsk state pedagogical university, Omsk, Russia Omsk state pedagogical university Omsk Russia

**Keywords:** terrestrial oligochaetes, soil macroinvertebrates, macrofauna, detritivores, species diversity, population density, community composition

## Abstract

**Background:**

Since the late 1980s, long-term monitoring of terrestrial ecosystems in metal-contaminated areas has been carried out in the Central Urals. As a part of these monitoring programmes, the data on soil macroinvertebrates in undisturbed areas as reference sites continues to be gathered. These data help study the local biodiversity and long-term dynamics of soil macroinvertebrate abundance in non-polluted areas.

**New information:**

The dataset (available from the GBIF network at https://www.gbif.org/dataset/bf5bc7f6-71a3-4abd-8abc-861ee3cbf84a) includes information from a long-term monitoring programme for two taxa of Annelids, Lumbricidae and Enchytraeidae, which dwell in the topsoil of spruce-fir, birch, pine and floodplain forests in the Central Urals. The dataset includes information on the earthworm community structure (list of species, species abundance, number of egg cocoons, cocoon exuvia, juveniles and adults) and enchytraeid abundance. The dataset consists of 553 sampling events (= samples, corresponding to upper and lower layers of the soil monoliths) and 12739 occurrences (earthworms, mainly identified to species and earthworm cocoons and enchytraeids, identified to family) collected during 1990–1991, 2004, 2014–2016 and 2018–2020. In total, 3305 individuals of earthworms were collected, representing ten (out of twelve) species and all eight genera recorded for the fauna of the Central Urals. In addition, 7292 earthworm egg cocoons and cocoon exuvia and 6926 individuals of enchytraeids were accumulated. The presence-absence data on each of the ten earthworm species, egg cocoons, cocoon exuvia and enchytraeids are provided for each sampling event. All data were collected in undisturbed non-polluted areas and are used as a local reference for ecotoxicological monitoring. The dataset provides valuable information for estimating the composition and abundance of earthworm communities in different habitats over a long time and contributes to the study of soil fauna biodiversity in the Urals.

## Introduction

Earthworms (Lumbricidae) are generally recognised as ecosystem engineers in temperate and tropical climates; they affect soil structure, food webs and nutrient cycles (Lavelle et al. 1997, Lavelle et al. 2006). Earthworms, amongst other macrodetritivores, largely determine the rate of organic matter decomposition and plant provision with nutrients, contributing to soil structure formation, thereby influencing soil water regime and fertility and modifying the microflora composition (Brussaard et al. 2007). Given such a significant role, earthworms and other annelids are often used in environmental monitoring (Paoletti et al. 2010) and pollution controls (Cortet et al. 1999).

The presented dataset includes information on annelid abundance and community composition in forests of the Central Urals. Other macroinvertebrates were collected, but not considered in this research. In the study area, two taxa of annelids – earthworms and enchytraeids – are the main soil macrodetritivores. Other groups of macrodetritivores are low-abundant (diplopods) or occasional (woodlice, wood cockroaches *Ectobius* spp.) compared to western European or more southern regions. Nematoceran larvae (Tipulidae, Limoniidae, Bibionidae, Sciaridae, Chironomidae, Cecidomyiidae and others), Coleopteran larvae (Elateridae) and molluscs are classified as phytosaprophages and their abundance is lower than annelids.

The study of earthworms in the Urals was started at the beginning of the 20^th^ century. In 1901, Wilhelm Michaelsen mentioned the first single find of earthworms in the Urals (Michaelsen 1901). He described *Eiseniaintermedia* (Michaelsen, 1901) based on one specimen from southern Bashkiria, referring it to the genus *Dendrobaena*. In 1950, Josef Malevich described two more endemics of the Urals, *Eiseniauralensis* Maleviĉ, 1950 and *Allobophorabasckirica* Maleviĉ, 1950 (Malevich 1950). The latter became a synonym for *Eiseniellatuberosa* Svetlov, 1924, later renamed *Pereliatuberosa* (Svetlov 1924). Thus, the first report on earthworm fauna in the Urals included 12 species (Malevich 1954), with three endemics (*Eiseniaintermedia*, *E.uralensis* and *P.tuberosa*). In 1967, Tamara Perel described the fourth endemic of the Urals, *Rhiphaeodrilusdiplotetratheca* (Perel, 1967), as a subspecies of *Allolobophorahandlirschi* (Perel 1967). Later, *Rhiphaeodrilus* was re-described as the monospecies genus (Csuzdi and Pavlíček 2005).

In summary, Tamara Perel (Perel 1979) described the fauna of the Urals as follows: almost exclusively endemic species *R.diplotetratheca*, *E.intermedia* and *P.tuberosa* are widespread in uncultivated soils; *E.uralensis* is characteristic for floodplain biotopes, while *Eisenianordenskioldi* (Eisen, 1873) is very rare. All other species are peregrine, they are occasional or occur near settlements: *Aporrectodeacaliginosa* (Savigny, 1826), *Aporrectodearosea* (Savigny, 1826), *Bimastosrubidus* (Savigny, 1826), *Dendrobaenaoctaedra* (Savigny, 1826), *Eiseniafetida* (Savigny, 1896), *Eiseniellatetraedra* (Savigny, 1826), *Octolasionlacteum* (Örley, 1885), *Lumbricusterrestris* Linnaeus, 1758 and *Lumbricusrubellus* Hoffmeister, 1843. Therefore, in terms of species richness and endemicity, the earthworm fauna of the Urals are much lower than the highly diverse and endemic fauna of more southern mountain ranges, Caucasus and Altai (Perel 1979).

From biogeography, the Urals are divided into five parts, the Southern, Central, Northern, Subpolar and Polar (Borisevich et al. 1968, Fig. 1). By the maps of the distribution of earthworm species in the north of the Palaearctic, the following species are recorded for the Central Urals (Vsevolodova-Perel 1988): *R.diplotetratheca*, *E.nordenskioldi*, *E.atlavinyteae* Perel & Graphodatsky, 1984 (this species was isolated from the previous one (Perel and Grafodatskiy 1984)), *O.lacteum*, *D.octaedra*, *B.rubidus* and *E.tetraedra*. According to the Cadastre of Earthworms of the Fauna of Russia, *A.caliginosa*, *A.rosea* and *E.fetida* may inhabit the Central Urals (Vsevolodova-Perel 1997). Studies in the fir-spruce forests in the Central Urals (Vorobeichik 1998) supplemented this list with two more species, *P.tuberosa* and *L.rubellus*. Fieldworks in the fir-spruce forests in the Pechoro-Ilychsky Biosphere Reserve (Northern Urals) confirmed the habitation of all species, except for *P.tuberosa* and *E.tetraedra* (Geraskina 2017).

In summary, the species richness of earthworms in the Central Urals (12 species) is greater than in the Northern Urals (10 species), the Subpolar Urals (three species) and the Polar Urals (four species), but less than in the Southern Urals (15 species) (Vsevolodova-Perel 1988, Ermakov and Golovanova 2010, Geraskina 2017, Makarova and Kolesnikova 2019). Furthermore, the percentage of endemic species (2 out of 12, 17%) is lower than in the Southern Urals (4 out of 15, 26%). The undisturbed habitats are dominated by *R.diplotetratheca* and *P.tuberosa*. By origin, except endemics, *E.nordenskioldi* and *E.atlavinyteae* are Siberian species and other species are peregrine.

In the study area, earthworms can be divided into three ecological categories (according to Bouché 1977): epigeic, epi-endogeic and endogeic. Anecic earthworms, typical for the more western European regions (e.g. *Lumbricusterrestris* or *Aporrectodealonga* (Ude, 1885)), are absent. Epigeic species feeding on the plant litter and inhabiting only the O horizon are represented by *D.octaedra* and *B.rubidus*. Epi-endogeic species dwelling in the O horizon and the upper (0–10 cm) layer of A horizon are *R.diplotetratheca*, *L.rubellus* and *E.atlavinyteae*. Endogeic species feeding on soil organic matter in the middle (10–20 cm) of mineral horizons are represented by *A.rosea*, *P.tuberosa* and *O.lacteum*. In coniferous forests, epi-endogeic species dominate (70–80% on density, mainly *R.diplotetratheca*) and endogeic species are of comparable abundance in deciduous forests. In the meadows, these endogeic species are accompanied by *A.caliginosa*, which dwell in mineral layers deeper than 20 cm.

The presented dataset includes ten species belonging to eight genera of the family Lumbricidae. Two species are absent: *E.nordenskioldi* and *E.fetida*. The first species is typical for the Cis-Urals and Trans-Urals (Perel 1979) and more northern areas of the Central Urals (Vorobeichik 1998). The second one is mainly inhabiting meadows, pastures and other biotopes with manure-amended soils; this species was also recorded in the study area, but outside the forest biotopes.

Enchytraeids range from 0.1–0.5 mm to 10–20 mm, i.e. they occupy an intermediate position between mesofauna and macrofauna. Gongalsky (Gongalsky 2021) pointed out that often “soil zoologists use the taxonomic, but not the dimensional principle to attribute a group to either the meso- or macrofaunal groups.” Therefore, enchytraeids are often referred to as mesofauna. We do not have data on enchytraeid abundance with extraction by the wet-funnel technique. The density of hand-sorted enchytraeids, i.e. individuals over 1–2 mm, wildly underestimates taxon abundance. Nevertheless, the numbers of large individuals can be used as a density index correlating with the taxon abundance. In addition, it would be wrong to deliberately exclude enchytraeids with a maximal possible size of about 10–20 mm from consideration since this can lead to biases in soil macrofauna investigation.

Unfortunately, we do not have data on the species composition of enchytraeids in the Urals. There were no specialists in this taxon for a long time in Russia and the country’s territory was almost a blank spot (Nurminen 1980). The situation began to improve only recently (Degtyarev et al. 2020), but so far, the fauna of the Urals has not been studied at all.

Russia is often a blank spot in global biodiversity databases and the global earthworm database is no exception (Phillips et al. 2019). Although Russia comprises 12.7% of the world’s land (excluding Antarctica), only 1.7% of research sites (179 out of 10842) are included in this database from its territory; all of them are in the European part, not including the Urals. Such a geographic bias can influence the analysis of global patterns. In the Global Biodiversity Information Facility (GBIF), the number of earthworm occurrences from Russia is comparable to that of other countries. However, specialised datasets (Shashkov et al. 2019) and occurrences of earthworms in datasets on soil invertebrates (Konakova and Kolesnikova 2021, Konakova et al. 2021, Rybalov and Tikhomirova 2021) are few. Moreover, most of the occurrences are concentrated in one dataset (6926 out of 10563 total occurrences) (Shashkov and Ivanova 2021).

The presented dataset includes information on several years within three decades. Such long-term studies provide the most comprehensive information on the local abundance and community composition of soil animals. This information is essential for several reasons. First, combined with data on the weather conditions, the dataset can be used to analyse potential climate change effects on earthworms (Singh et al. 2019). Second, estimating the spatial and temporal variation in soil animal density is necessary to determine sampling efforts and plan the correct sampling design. Moreover, the before-mentioned variation must be assessed at two spatial scales, within sampling plots and study sites. Third, combined with habitat characteristics, the dataset can be used to analyse factors affecting earthworm abundance and diversity.

## Project description

### Study area description

**Figure 1.**
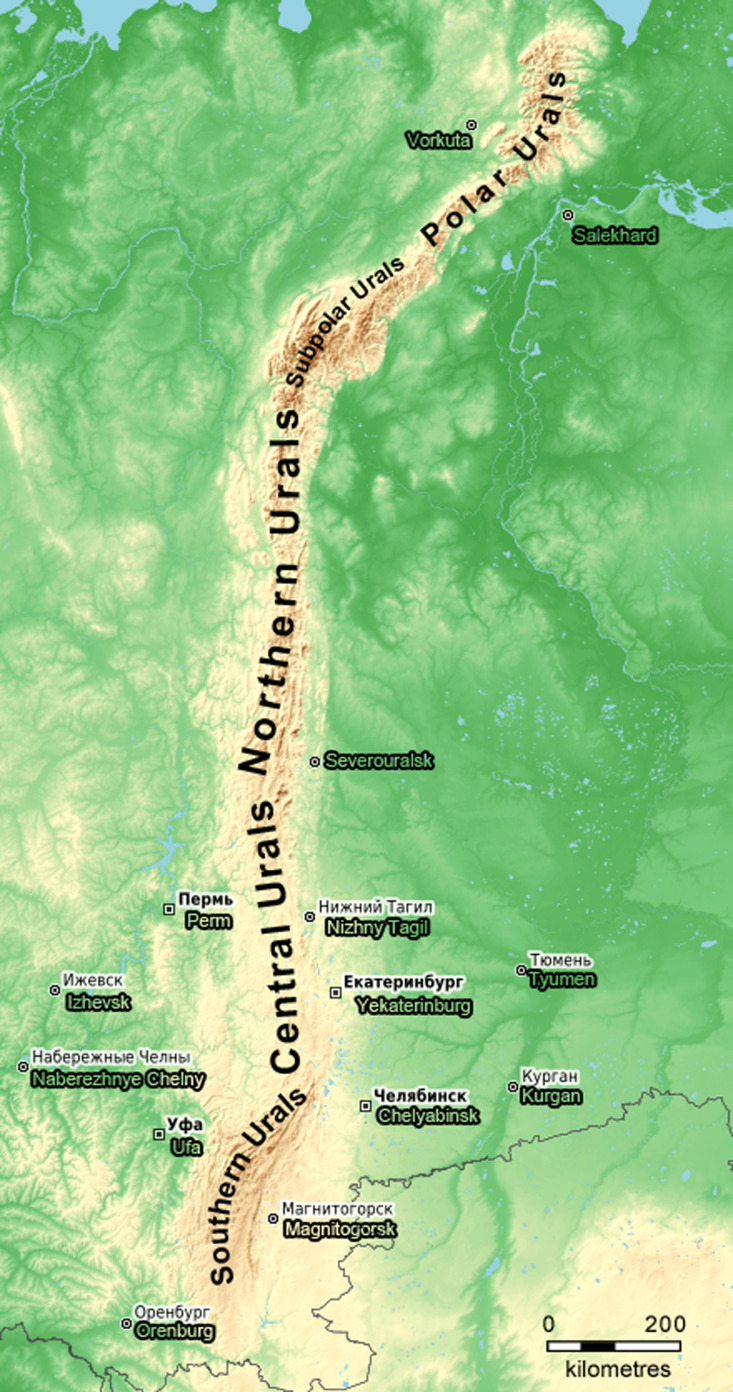
A scheme of biogeographic division of the Ural mountain range, based on the data from Open Street Map (OpenStreetMap contributors 2017).

The Ural Mountains are a north-south-orientated mountain system, located between the East European plain and West Siberian plain (Fig. 1). The study area is situated in the lowest uplands of the Urals (altitudes are 150–400 m above sea level) and belongs to the southern taiga subzone (Kulikov et al. 2013, Fig. 2). Primary coniferous forests (*Piceaabies* (L.) H.Karst., *Abiessibirica* Ledeb. and *Pinussylvestris* L.) and secondary deciduous forests (*Betulapendula* Roth, *Betulapubescens* Ehrh. and *Populustremula* L.) prevail. Spruce and fir forests with nemoral flora on loam or heavy loam soils dominate on the western slope of the Urals and pine forests on sandy loam or light loam soils prevail on the eastern side (Kulikov et al. 2013). The ground vegetation layer is dominated by *Oxalisacetosella* L., *Aegopodiumpodagraria* L., *Gymnocarpiumdryopteris* (L.) Newman, *Dryopteriscarthusiana* (Vill.) H.P.Fuchs, *Asarumeuropaeum* L., *Maianthemumbifolium* (L.) F.W.Schmidt, *Cerastiumpauciflorum* Stev. ex Ser. and *Rabeleraholostea* (L.) M.T.Sharples & E.A.Tripp.

Soil formation occurs on eluvium and eluvium-diluvium of bedrock metamorphic rocks (shales, sandstones, quartzites and silicified limestones). Soil cover is formed mainly by soddy-podzolic soils (Albic Retisols, Stagnic Retisols and Leptic Retisols), burozems (Haplic Cambisols) and grey forest soils (Retic Phaeozems) (Kaigorodova and Vorobeichik 1996). Zoogenically-active humus form (Dysmull) prevail (Korkina and Vorobeichik 2016, Korkina and Vorobeichik 2018, Korkina and Vorobeichik 2021).

The climate is Warm Summer Humid Continental, "Dfb" according to the Köppen-Geiger classification (Peel et al. 2007). The average annual air temperature is +2.0°С; the average annual precipitation is 550 mm; the warmest month is July (+17.7°С) and the coldest month is January (–14.2°С) (mean values for the last 40 years, 1975–2015, according to the data of the nearest meteorological station in Revda). The snowless period is about 215 days (from April to October), the maximum depth of the snow cover is about 40–60 cm.

## Sampling methods

### Study extent

Study sites were located on gentle slopes of ridges in forests with a different stand composition (spruce-fir, pine and birch forests) and arable lands. Loam and heavy loam soddy-podzolic soils (Albic Retisols and Stagnic Retisols) prevail (Table 1).

**Figure 2.**
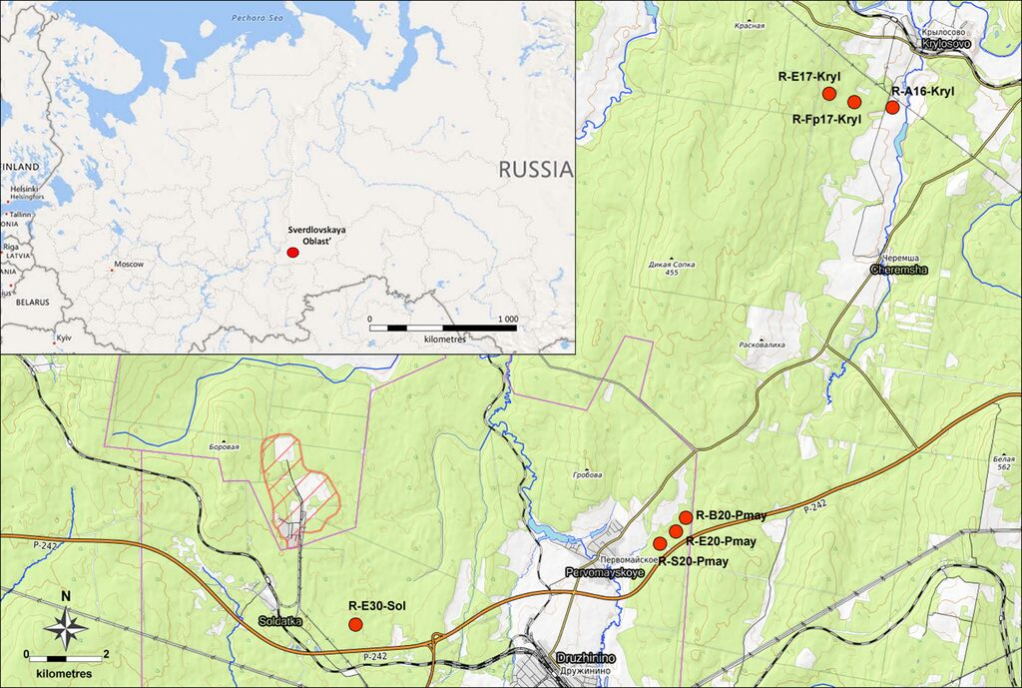
Location of the study sites (= LocationID) in the Central Urals (a scheme based on the data from Open Street Map (OpenStreetMap contributors 2017)).

A total of seven study sites (= dwc:locationID) were established corresponding to local aggregations of different biotopes (Fig. 2). The number of sampling plots within each study site were unequal: R-E30-Sol (spruce-fir forest) included seven sampling plots, R-E20-Pmay (spruce-fir forest) included six plots, R-B20-Pmay (birch forest) and R-S20-Pmay (pine forest) included one sampling plot each, R-E17-Kryl (spruce and birch forests) included four plots, R-Fp17-Kryl (floodplain forest) and R-A16-Kryl (arable land) included three plots each.

Study sites R-E30-Sol and R-E20-Pmay were permanent throughout all years of the study (Table 2). However, the exact position of the sampling plots within these study sites differed between 1990–1991 and 2004–2020 (exact position varied within a range of 300–500 m due to refinement of methodical procedures and positioning inaccuracy). The current position of the sampling plots has been established since 2004. Study sites R-B20-Pmay and R-S20-Pmay are additional and were included in the study only in 1991. Study sites near Krylosovo (R-E17-Kryl, R-Fp17-Kryl and R-A16-Kryl) have been explored since 2019.

The study of earthworms is part of an ongoing long-term monitoring project, which currently covers the following years: 1990 (12 June), 1991 (12 June – 14 July), 2004 (04 July – 16 August), 2014 (02 July – 20 August), 2015 (06 August – 01 September), 2016 (21 July – 11 August), 2018 (16 July – 05 August), 2019 (19 June – 11 August) and 2020 (12 July – 17 July).

### Sampling description

Earthworms were collected in June, July and August from 1990–2020. Sampling plots 10 × 10 m in size were established in seven study sites (Table 2).

**Figure 3.**
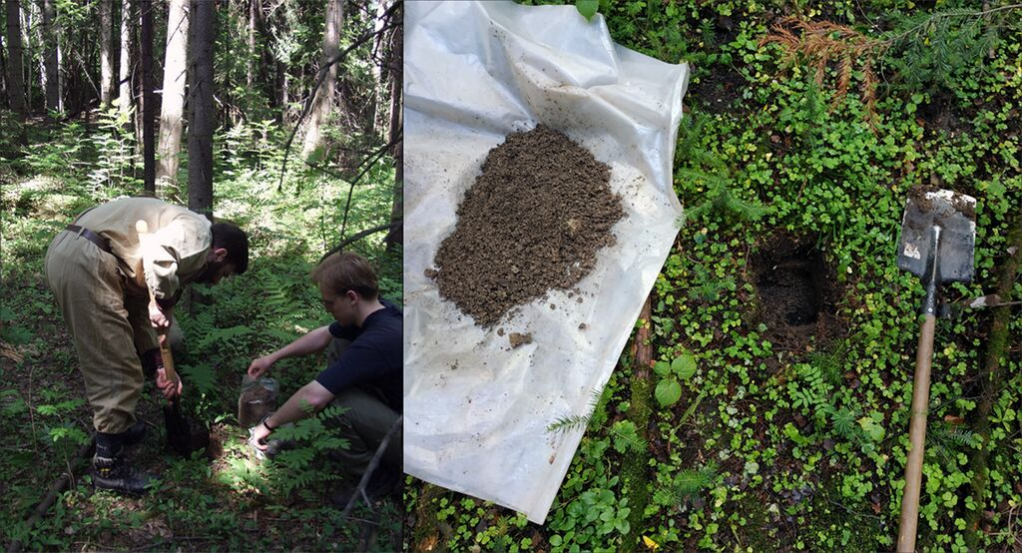
The process of sampling.

Annelids (earthworms and enchytraeids) were hand-sorted out of soil monoliths 20 × 20 cm in area and 25–30 cm in depth, depending on the occurrence of macroinvertebrates (Fig. 3). The time interval for extracting one soil monolith from the sampling plot was approximately 5 minutes. In most cases, ten monoliths were collected from each plot, except for one monolith from R-E30-Sol in 2020; two monoliths from R-E17-Kryl, R-Fp17-Kryl and R-A16-Kryl in May 2019 and R-E30-Sol in 2020; three monoliths from R-E17-Kryl, R-Fp17-Kryl and R-A16-Kryl in August 2019; five monoliths from R-E20-Pmay in 2015 and 2016 and R-E30-Sol in August 2016 and 2018; 11 monoliths from R-E30-Sol in August 2015; 40 monoliths from R-E20-Pmay in 1990 (Table 2). The monoliths were collected randomly, excluding nearby trunk areas with a radius of 0.5–1 m around large trees (more than 30 cm in diameter) and any visible pedoturbations. During sampling, each monolith was divided into two layers, corresponding to the samples: the O horizons (forest litter) and A horizon (organic-mineral). Monoliths were not subdivided into layers and were analysed as a whole sample (the A horizon) in R-A16-Kryl (arable land, see Table 2). Monoliths were placed in plastic bags (separately for the layers), delivered to the laboratory and stored before processing at 12°C for no more than five days (as a rule, 1–2 days). The collected earthworms were carefully washed with water, fixed with 10% formalin and then wet-preserved in 70% ethanol. Enchytraeids and earthworm cocoons were fixed with 70% ethanol.

**Figure 4.**
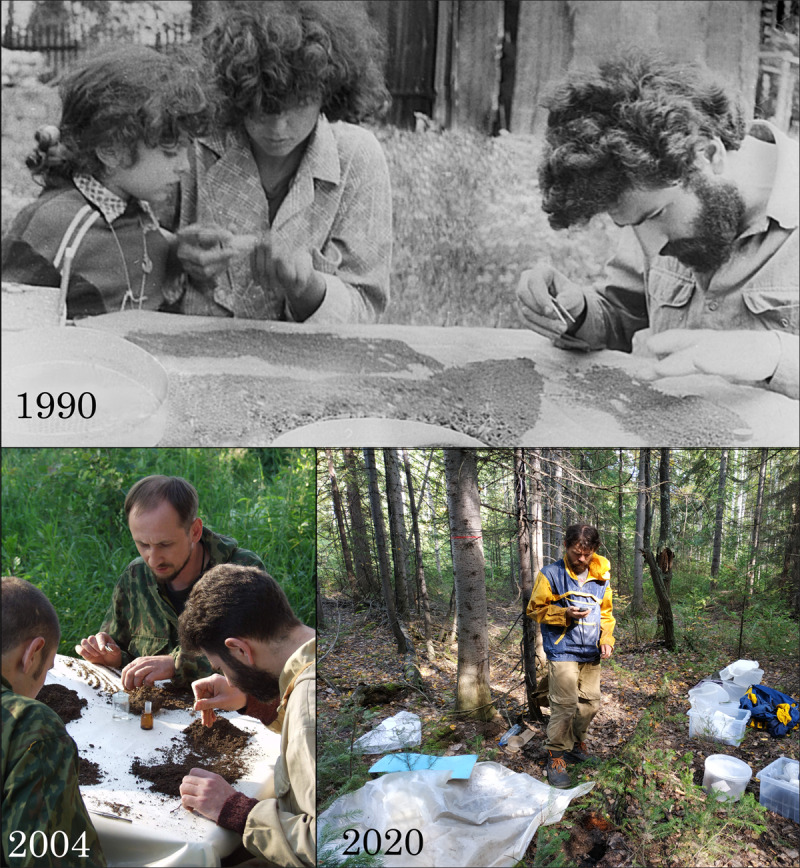
Studying soil macrofauna for over 30 years (photos from the personal archives of the authors).

The sampling and hand sorting procedures were the same in all years. Thus, a total of 284 soil monoliths and 553 samples (organic and organic-mineral horizons) were collected over all these years (Fig. 4).

Unfortunately, the materials collected in 1990 and 1991 were not preserved in full until now. Therefore, in the dataset, unlike all others, these years marked with dwc:basisOfRecord = "HumanObservation."

### Quality control

A total of more than 3300 individuals of earthworms, 7200 egg cocoons and cocoon exuvia of earthworms and 6900 individuals of enchytraeids were collected. All specimens were wet-preserved in 70% alcohol and stored (with the partial exception of materials from 1990–1991) in the depository of the Laboratory of Population and Community Ecotoxicology of the Institute of Plant and Animal Ecology, Ural Branch, Russian Academy of Sciences (IPAE UB RAS). Adult earthworms were identified to species level using the taxonomic key for the fauna of Russia (Vsevolodova-Perel 1997). Juvenile specimens were identified to species level using external characteristics, such as the colouration, the prostomium shape, the pattern of setae and examination of the internal structure during autopsy (the shape of nephridial bladders, the presence and location of diverticula). Almost all earthworms (3236 of 3305, 98%) were identified to species level. Earthworm cocoons and enchytraeids were identified only to the family level. Earthworms were identified by Evgenii Vorobeichik and Dina Nesterkova from IPAE UB RAS and Elena Golovanova from the Laboratory of Invertebrate Systematics and Ecology of Omsk State Pedagogical University.

## Geographic coverage

### Description

The study area is located in the southern taiga subzone of the Central Urals, 60–70 km westwards from Yekaterinburg. Study sites are placed in coniferous forests (spruce-fir and pine), secondary birch forests, floodplain forests of small rivers and cultivated arable lands.

### Coordinates

56.789 and 56.957 Latitude; 59.33 and 59.745 Longitude.

## Taxonomic coverage

### Description

General taxonomic coverage is one phylum, one class, two orders, two families, eight genera and ten species of annelids.

### Taxa included

**Table:** 

Rank	Scientific Name	
class	Clitellata	
order	Crassiclitellata	
family	Lumbricidae	earthworms
order	Enchytraeida	
family	Enchytraeidae	pot worms

## Temporal coverage

**Formation period:** 1990-2020.

### Notes

At present, the following period is covered: 12 June 1990 – 17 July 2020.

## Collection data

### Collection name

lepc_annelids_1990-2020

### Specimen preservation method

alcohol, formalin

## Usage licence

### Usage licence

Other

### IP rights notes

This work is licensed under a Creative Commons Attribution (CC-BY) 4.0 Licence.

## Data resources

### Data package title

Long-term dynamics of the abundance of earthworms and enchytraeids (Annelida, Clitellata: Lumbricidae, Enchytraeidae) in forests of the Central Urals, Russia

### Resource link

https://www.gbif.org/dataset/bf5bc7f6-71a3-4abd-8abc-861ee3cbf84a

### Number of data sets

1

### Data set 1.

#### Data set name

Long-term dynamics of the abundance of earthworms and enchytraeids (Annelida, Clitellata: Lumbricidae, Enchytraeidae) in forests of the Central Urals, Russia

#### Data format

Darwin Core

#### Number of columns

41

#### Download URL

http://gbif.ru:8080/ipt/archive.do?r=lepc_annelids_1990-2020&v=1.2

#### Data format version

1.2

#### Description

The dataset (Vorobeichik et al. 2021) presents information from a long-term monitoring programme for two taxa of Annelids, Lumbricidae and Enchytraeidae, which dwell in the topsoil of spruce-fir, birch, pine and floodplain forests in the Central Urals. The dataset describes the earthworm community structure (list of species, species abundance, number of egg cocoons, cocoon exuvia, juveniles and adults) and enchytraeid abundance. The dataset consists of 553 sampling events (= samples), corresponded to 12739 occurrences (earthworms, mainly identified to species and earthworm cocoons and enchytraeids, identified to family), collected during 1990–1991, 2004, 2014–2016 and 2018–2020. In total, 3305 individuals of earthworms were collected, representing ten (out of twelve) species and all eight genera recorded for the fauna of the Central Urals. In addition, 7292 earthworm egg cocoons and cocoon exuvia and 6926 individuals of enchytraeids were accumulated. The presence-absence data on each of the ten earthworm species, egg cocoons, cocoon exuvia and enchytraeids are provided for each sampling event. All data were collected in undisturbed non-polluted areas and are used as a local reference for ecotoxicological monitoring. The dataset provides valuable information for estimating the composition and abundance of earthworm communities in different habitats over a long time and contributes to the study of soil fauna biodiversity in the Urals.

**Data set 1.** 

Column label	Column description
eventID	An identifier for the set of information associated with an Event, constructed from designations of the year, study area, number of the sampling plot, number of the sample and designation of the soil layer. May contain additional information. A variable. Example: "R2004-E30-2-Sol-13L".
occurrenceID	An identifier for the Occurrence. Constructed from a combination of dwc:eventID and the number of occurrence within the suggested event. A variable. Example: "R2004-E20-15-Pmay-143L-18".
eventDate	The sampling date in the "year-month-day" format. A variable. Example: "2004-07-04".
habitat	A category of the habitat in which the Event occurred. Contains data on the vegetation community and soil description of the sampling plots. A variable. Example: "Abietum oxalidosum on Albic Retisol".
lifeStage	The age class or life stage of the earthworms at the time the Occurrence was recorded. A variable. Examples: "adult", "juvenile", "cocoon".
occurrenceRemarks	Comments or notes about the Occurrence. A state of the cocoons. A variable. Examples: "egg cocoon", "cocoon exuvium".
basisOfRecord	The specific nature of the data record. A constant "PreservedSpecimen".
decimalLatitude	The geographic latitude (in decimal degrees, using the spatial reference system given in geodeticDatum) of the geographic centre of the sampling plot. A variable. Example: "56.7210".
decimalLongitude	The geographic longitude (in decimal degrees, using the spatial reference system given in geodeticDatum) of the geographic centre of the sampling plot. A variable. Example: "59.4280".
coordinateUncertaintyInMetres	The horizontal distance (in metres) from the given decimalLatitude and decimalLongitude describing the smallest circle containing the whole of the Location. A variable. Examples: "10", "100".
geodeticDatum	The ellipsoid, geodetic datum or spatial reference system (SRS) upon which the geographic coordinates given in decimalLatitude and decimalLongitude are based. A constant "WGS84".
stateProvince	The name of the next smaller administrative region than country (state, province, canton, department, region etc.) in which the Location occurs. A constant "Sverdlovskaya Oblast'".
municipality	The full, unabbreviated name of the next smaller administrative region than county (city, municipality, etc.) in which the Location occurs. A variable. Example: "Nizhniye Sergi".
locality	The specific description of the place. Less specific geographic information can be provided in other geographic terms (higherGeography, continent, country, stateProvince, county, municipality, waterBody, island, islandGroup). A variable. Example: "Pervomayskoye".
locationID	An identifier for the set of location information (data associated with dcterms:Location), corresponding to the study sites. A variable. Example: "R-E20-Pmay".
organismQuantity	A number value for the quantity of organisms.
organismQuantityType	The type of quantification system used for the quantity of organisms. A constant "individuals".
samplingProtocol	The description of the method or protocol used during an Event. A constant "extraction of soil monoliths followed by hand-sorting in laboratory".
samplingEffort	The amount of effort expended during an Event. A constant "284 soil monoliths in total; 10 monoliths randomly extracted from 10 x 10 m plot on 7 study sites and 25 sampling plots".
sampleSizeValue	A numeric value for a measurement of the size of a sample in a sampling event. A constant "20 L x 20 W x 25–30 D".
sampleSizeUnit	The unit of measurement of the size of a sample in a sampling event. A constant "centimetres".
occurrenceStatus	A statement about the presence or absence of a Taxon at a Location. A variable. Examples: "present", "absent".
scientificName	The full scientific name, with authorship and date information. A variable. Example: "Dendrobaenaoctaedra (Savigny, 1826)".
scientificNameAuthorship	The authorship information for the scientificName formatted according to the conventions of the applicable nomenclaturalCode. A variable. Example: "(Savigny, 1826)".
kingdom	The full scientific name of the kingdom in which the taxon is classified. A constant "Animalia".
phylum	The full scientific name of the phylum or division in which the taxon is classified. A constant "Annelida".
class	The full scientific name of the class in which the taxon is classified. A constant "Clitellata".
order	The full scientific name of the order in which the taxon is classified. A variable. Example: "Crassiclitellata".
family	The full scientific name of the family in which the taxon is classified. A variable. Example: "Lumbricidae".
genus	The full scientific name of the genus in which the taxon is classified. A variable. Example: "Dendrobaena".
specificEpithet	The name of the first or species epithet of the scientificName. A variable. Example: "octaedra".
taxonRank	The taxonomic rank of the most specific name in the scientificName. A variable. Example: "species".
year	The four-digit year in which the Event occurred, according to the Common Era Calendar. A variable. Example: "2004".
month	The ordinal month in which the Event occurred. A variable. Example: "7".
recordedBy	A list (concatenated and separated) of names of people responsible for recording the original Occurrence. A variable. Example: "Maxim E. Grebennikov | Petr G. Pishchulin | Evgenii L. Vorobeychik".
identifiedBy	A list (concatenated and separated) of names of people who assigned the Taxon to the subject. A variable. Example: "Elena V. Golovanova".
country	The name of the country in which the Location occurs. A constant "Russian Federation".
countryCode	The standard code for the country in which the Location occurs. A constant "RU".
ownerInstitutionCode	The name (or acronym) in use by the institution having ownership of the object(s) or information referred to in the record. A constant "Institute of Plant and Animal Ecology (IPAE)".
institutionCode	The name (or acronym) in use by the institution having custody of the object(s) or information referred to in the record. A constant "Institute of Plant and Animal Ecology (IPAE)".
dynamicProperties	A list of additional measurements, facts, characteristics or assertions about the record. The soil layer in which the sample was collected. A variable. Example: "{"soilHorizon":"O"}".

## Figures and Tables

**Table 1.** Characteristics of the sampling plots. Soil description is given according to WRB 2015. Soil pH is given as mean (standard deviation for n = 5); the asterisk denotes data, based on one sample (taken from soil profile). Soil texture: SL – sandy loam, ML – medium loam, HL – heavy loam, C – clay.

Study site (dwc: locationID)	Sampling plot (Refers to dwc: eventID)	Decimal latitude	Decimal longitude	Soil description	Soil texture of A horizon / lower part of the soil profile	pH (water)		Vegetation
						O horizon	A horizon	
R-E30-Sol	1 (R{year}-E30-1…)	56.8013	59.4249	Albic Retisol	ML / HL	5.7 (0.2)	4.9 (0.2)	Abietum oxalidosum
	2 (R{year}-E30-2…)	56.7996	59.4276	Albic Retisol	ML / HL	5.6 (0.1)	4.9 (0.1)	Abietum oxalidosum
	3 (R{year}-E30-3…)	56.7988	59.4274	Stagnic Retisol	ML / HL	5.3 (0.2)	4.6 (0.2)	Abieto-Picietum oxalidosum
	4 (R2004-E30-4…)	56.7210	59.4280	Stagnic Retisol	ML / HL	5.1 (0.3)	4.9 (0.2)	Abieto-Picietum oxalidosum
	6 (R2020-E30-6…)	56.7985	59.4286	Stagnic Retisol	ML / HL	5.7*	4.8*	Abieto-Picietum oxalidosum
	7 (R2020-E30-7…)	56.8035	59.4299	Stagnic Retisol	ML / HL	5.5*	4.6*	Picieto-Abietum oxalidosum
	S. plot of 1991 (R1991-E30…)	56.800	59.450	Stagnic Retisol	ML / HL			Abieto-Picietum oxalidosum
R-B20-Pmay	S. plot of 1991 (R1991-B20…)	56.824	59.574	Stagnic Retisol	ML / HL	5.6 (0.2)	5.0 (0.1)	Betuletum herbosum
R-E20-Pmay	1 (R{year}-E20-1…)	56.8230	59.5728	Stagnic Retisol	ML / ML	4.9 (0.2)	4.2 (0.1)	Picietum oxalidosum
	2 (R2014-E20-2…)	56.8211	59.5762	Stagnic Retisol	ML / HL	5.5 (0.1)	4.4 (0.1)	Picieto-Abietum oxalidosum
	15 (R2004-E20-15…)	56.8240	59.5700	Stagnic Retisol	ML / ML			Picietum oxalidosum
	17 (R2004-E20-17…)	56.8210	59.5770	Stagnic Retisol	ML / HL			Picieto-Abietum oxalidosum
	S. plot of 1990 (R1990-E20…)	56.823	59.573	Stagnic Retisol	ML / ML			Picietum oxalidosum
	S. plot of 1991 (R1991-E20…)	56.823	59.573	Stagnic Retisol	ML / ML			Picietum oxalidosum
R-S20-Pmay	S. plot of 1991 (R1991-S20…)	56.820	59.566	Stagnic Retisol	ML / HL	5.0 (0.1)	4.6 (0.1)	Pineetum herbosum
R-E17-Kryl	3 (R2019-E17-3…)	56.9283	59.6517	Stagnic Retisol	ML / HL			Picieto-Abietum oxalidosum
	6 (R2019-E17-6…)	56.9286	59.6462	Stagnic Retisol	ML / HL	5.1 (0.1)	4.3 (0.2)	Picietum oxalidosum
	12 (R2019-E17-12…)	56.9310	59.6477	Endocalcaric Luvisol	HL / C	5.6 (0.1)	5.3 (0.2)	Betuletum oxalidosum
	35 (R2019-E17-35…)	56.9278	59.6522	Stagnic Retisol	ML / HL	5.4 (0.2)	4.4 (0.2)	Betuletum oxalidosum
R-Fp17-Kryl	5 (R2019-Fp17-5…)	56.9294	59.6462	Fluvic Umbriс Gleysol	ML / HL	5.7 (0.1)	5.8 (0.5)	Alnetum incanae herbosum
	20 (R2019-Fp17-20…)	56.9287	59.6492	Fluvic Umbriс Gleysol	ML / SL	5.5 (0.1)	5.4 (0.2)	Betuletum herbosum
	38 (R2019-Fp17-38…)	56.9293	59.6536	Fluvic Gleyic Umbrisol	ML / C	5.7 (0.2)	6.2 (0.3)	Alnetum incanae herbosum
R-A16-Kryl	98 (R2019-A16-98…)	56.9286	59.6640	Albic Retisol (Aric)	ML / HL	5.7 (0.2)	5.8 (0.1)	Agricultural crops
	102 (R2019-A16-102…)	56.9294	59.6685	Albic Retisol (Aric)	ML / ML	5.6 (0.2)	6.1 (0.1)	Agricultural crops
	114 (R2019-A16-114…)	56.9318	59.6700	Albic Retisol (Aric)	ML / HL	5.4 (0.1)	5.5 (0.1)	Agricultural crops

**Table 2.** Total number of the sampling plots (soil monoliths\samples) at the study area.

Year	Month	Study site						
		R-E30-Sol	R-E20-Pmay	R-B20-Pmay	R-S20-Pmay	R-E17-Kryl	R-Fp17-Kryl	R-A16-Kryl
1990	June		1 (40\80)					
1991	June	1 (10\20)	1 (10\20)		1 (10\20)			
	July			1 (10\20)				
2004	July	3 (30\60)						
	August		2 (20\40)					
2014	July	2 (20\40)	1 (10\20)					
	August	1 (10\20)	1 (10\20)					
2015	August	1 (11\22)	1 (5\10)					
	September		1 (5\10)					
2016	July	1 (5\10)	1 (5\10)					
	August	1 (5\10)	1 (5\10)					
2018	July	2 (10\20)						
	August	1 (5\10)						
2019	June					1 (2\4)	1 (2\4)	1 (2\2)
	July					1 (10\20)	1 (10\20)	1 (10\10)
	August					1 (3\6)	1 (3\6)	1 (3\3)
2020	July	1 (2\4)				1 (1\2)		
**Total**		**7 (108\216)**	**6 (110\220)**	**1 (10\20)**	**1 (10\20)**	**4 (16\32)**	**3 (15\30)**	**3 (15\15)**

## References

[B7542381] Borisevich D. V., Vendrov S. L., Gorchakovskiy P. L., Zernova L. I., Kemmerikh A. O., Kirikov S. V., Klyukanova I. A., Komar I. V., Kuashinova K. V., Pogodina G. S., Privalovskiy G. A., Rozov N. N., Chikishev A. G. (1968). Ural i Priural'ye.

[B7543381] Bouché M. B., Lohm U., Persson T. (1977). Soil organisms as components of ecosystems.

[B7466829] Brussaard Lijbert, Pulleman Mirjam M., Ouédraogo Élisée, Mando Abdoulaye, Six Johan (2007). Soil fauna and soil function in the fabric of the food web. Pedobiologia.

[B7466818] Cortet Jérôme, Vauflery Annette Gomot-De, Poinsot-Balaguer Nicole, Gomot Lucien, Texier Christine, Cluzeau Daniel (1999). The use of invertebrate soil fauna in monitoring pollutant effects. European Journal of Soil Biology.

[B7467141] Csuzdi C., Pavlíček T. (2005). Earthworms from Israel. II. Remarks on the genus Perelia Easton, 1983 with description of a new genus and two new species.. Acta Zoologica Academiae Scientiarum Hungaricae.

[B7467150] Degtyarev Maksim I., Lebedev Iurii M., Kuznetsova Ksenia G., Gongalsky Konstantin B. (2020). A history of study and new records of terrestrial enchytraeids (Annelida, Clitellata, Enchytraeidae) from the Russian Far East. ZooKeys.

[B7543331] Ermakov A. I., Golovanova E. V. (2010). Species composition and abundance of earthworms in the tundra biocenoses of Denezhkin Kamen’ Mountain (Northern Urals). Contemporary Problems of Ecology.

[B7466839] Geraskina A. P. (2017). The population of earthworms (Lumbricidae) in the main types of dark coniferous forests in Pechora-Ilych Nature Reserve. Biology Bulletin.

[B7467159] Gongalsky Konstantin B. (2021). Soil macrofauna: Study problems and perspectives. Soil Biology and Biochemistry.

[B7466848] Kaigorodova S. Yu., Vorobeichik E. L. (1996). Changes in certain properties of grey forest soil polluted with emissions from a copper-smelting plant. Russian Journal of Ecology.

[B7545760] Konakova T., Kolesnikova A. (2021). Large soil invertebrates of coniferous forests along gradient of air pollution: temporal series of the data (Komi Republic). Dataset/Sampling event.

[B7545776] Konakova T., Kolesnikova A., Taskaeva A. (2021). Soil invertebrates occurrences in European North-East of Russia. Dataset/Occurrence.

[B7467168] Korkina Irina N., Vorobeichik Evgenii L. (2021). Non-typical degraded and regraded humus forms in metal-contaminated areas, or there and back again. Geoderma.

[B7466866] Korkina I. N., Vorobeichik E. L. (2016). The humus index: A promising tool for environmental monitoring. Russian Journal of Ecology.

[B7466857] Korkina I. N., Vorobeichik E. L. (2018). Humus Index as an indicator of the topsoil response to the impacts of industrial pollution. Applied Soil Ecology.

[B7546268] Kulikov P. V., Zolotareva N. V., Podgayevskaya Y. N. (2013). Endemichnyye rasteniya Urala vo flore Sverdlovskoy oblasti.

[B7467177] Lavelle P., Bignell D., Lepage M., Wolters V., Roger P., Ineson P., Heal O. W., Dhillion S. (1997). Soil function in a changing world: The role of invertebrate ecosystem engineers. European Journal of Soil Biology.

[B7467190] Lavelle P., Decaëns T., Aubert M., Barot S., Blouin M., Bureau F., Margerie P., Mora P., Rossi J. -P. (2006). Soil invertebrates and ecosystem services. European Journal of Soil Biology.

[B7529279] Makarova O. L., Kolesnikova A. A. (2019). Earthworms (Oligochaeta, Lumbricidae) in the tundra of Eastern Europe. Biology Bulletin.

[B7466875] Malevich I. I. (1950). Novyye i maloizvestnyye vidy dozhdevykh chervey v faune Yevropeyskoy chasti SSSR [New and little-known species of earthworms in the fauna of the European part of the USSR]. Doklady AN SSSR. Novaya seriya..

[B7466884] Malevich I. I. (1954). K faune maloshchetinkovykh chervey (Oligochaeta) Urala i Priural’ya [To the fauna of small-bristled worms (Oligochaeta) of the Urals and the Cis-Urals]. Uchenyye zapiski MGPI imeni Potemkina.

[B7466893] Michaelsen W. (1901). Oligochaeten der Zoologischen Museen zu St. Petersburg und Kiew. Izvestiya Imperatorskoy Akademii Nauk.

[B7467204] Nurminen M. (1980). Notes on the Enchytraeids (Oligochaeta) of the USSR. Annales Zoologica Fennica.

[B7543244] contributors OpenStreetMap Planet dump retrieved from https://planet.osm.org. https://planet.osm.org/.

[B7466902] Paoletti M. G., Bressan M., Edwards C. A. (2010). Soil invertebrates as bioindicators of human disturbance. Critical Reviews in Plant Sciences.

[B7467213] Peel M. C., Finlayson B. L., McMahon T. A. (2007). Updated world map of the Köppen-Geiger climate classification. Hydrology and Earth System Sciences.

[B7466911] Perel T. S. (1967). Dozhdevyye chervi (Lumbricidae) Yuzhnogo Urala [Earthworms (Lumbricidae) of the Southern Urals]. Zoologicheskiy Zhurnal.

[B7466920] Perel T. S. (1979). Rasprostraneniye i zakonomernosti raspredeleniya dozhdevykh chervey fauny SSSR.

[B7466928] Perel T. S., Grafodatskiy A. S. (1984). Novyye vidy roda Eisenia (Lumbricidae, Oligochaeta) i ikh khromosomnyye nabory [New species of the genus *Eisenia* (Lumbricidae, Oligochaeta) and their chromosome sets]. Zoologicheskiy Zhurnal.

[B7466937] Phillips Helen R. P., Guerra Carlos A., Bartz Marie L. C., Briones Maria J. I., Brown George, Crowther Thomas W., Ferlian Olga, Gongalsky Konstantin B., van den Hoogen Johan, Krebs Julia, Orgiazzi Alberto, Routh Devin, Schwarz Benjamin, Bach Elizabeth M., Bennett Joanne M., Brose Ulrich, Decaëns Thibaud, König-Ries Birgitta, Loreau Michel, Mathieu Jérôme, Mulder Christian, van der Putten Wim H., Ramirez Kelly S., Rillig Matthias C., Russell David, Rutgers Michiel, Thakur Madhav P., de Vries Franciska T., Wall Diana H., Wardle David A., Arai Miwa, Ayuke Fredrick O., Baker Geoff H., Beauséjour Robin, Bedano José C., Birkhofer Klaus, Blanchart Eric, Blossey Bernd, Bolger Thomas, Bradley Robert L., Callaham Mac A., Capowiez Yvan, Caulfield Mark E., Choi Amy, Crotty Felicity V., Crumsey Jasmine M., Dávalos Andrea, Diaz Cosin Darío J., Dominguez Anahí, Duhour Andrés Esteban, van Eekeren Nick, Emmerling Christoph, Falco Liliana B., Fernández Rosa, Fonte Steven J., Fragoso Carlos, Franco André L. C., Fugère Martine, Fusilero Abegail T., Gholami Shaieste, Gundale Michael J., López Mónica Gutiérrez, Hackenberger Davorka K., Hernández Luis M., Hishi Takuo, Holdsworth Andrew R., Holmstrup Martin, Hopfensperger Kristine N., Lwanga Esperanza Huerta, Huhta Veikko, Hurisso Tunsisa T., Iannone Basil V., Iordache Madalina, Joschko Monika, Kaneko Nobuhiro, Kanianska Radoslava, Keith Aidan M., Kelly Courtland A., Kernecker Maria L., Klaminder Jonatan, Koné Armand W., Kooch Yahya, Kukkonen Sanna T., Lalthanzara H., Lammel Daniel R., Lebedev Iurii M., Li Yiqing, Jesus Lidon Juan B., Lincoln Noa K., Loss Scott R., Marichal Raphael, Matula Radim, Moos Jan Hendrik, Moreno Gerardo, Morón-Ríos Alejandro, Muys Bart, Neirynck Johan, Norgrove Lindsey, Novo Marta, Nuutinen Visa, Nuzzo Victoria, Pansu Johan, Paudel Shishir, Pérès Guénola, Pérez-Camacho Lorenzo, Piñeiro Raúl, Ponge Jean-François, Rashid Muhammad Imtiaz, Rebollo Salvador, Rodeiro-Iglesias Javier, Rodríguez Miguel Á., Roth Alexander M., Rousseau Guillaume X., Rozen Anna, Sayad Ehsan, van Schaik Loes, Scharenbroch Bryant C., Schirrmann Michael, Schmidt Olaf, Schröder Boris, Seeber Julia, Shashkov Maxim P., Singh Jaswinder, Smith Sandy M., Steinwandter Michael, Talavera José A., Trigo Dolores, Tsukamoto Jiro, de Valença Anne W., Vanek Steven J., Virto Iñigo, Wackett Adrian A., Warren Matthew W., Wehr Nathaniel H., Whalen Joann K., Wironen Michael B., Wolters Volkmar, Zenkova Irina V., Zhang Weixin, Cameron Erin K., Eisenhauer Nico (2019). Global distribution of earthworm diversity. Science.

[B7545789] Rybalov L. B., Tikhomirova A. L. (2021). Soil invertebrates surveys from the Chronicle of Nature of the Prioksko-Terrasnyi Biosphere Reserve. Dataset/Sampling event.

[B7545750] Shashkov M., Bobrovsky M., Smirnova O. (2019). Earthworms population in old-growth taiga forests of Pechoro-Ilych State Nature Reserve. Dataset/Sampling event.

[B7467232] Shashkov M, Ivanova N (2021). Earthworm communities (Oligochaeta: Lumbricidae) in old-growth and young forests of protected areas of the Kaluga Oblast (European Russia). Dataset/ Samplingevent..

[B7467222] Singh Jaswinder, Schädler Martin, Demetrio Wilian, Brown George G., Eisenhauer Nico (2019). Climate change effects on earthworms - a review. Soil Organisms.

[B7467084] Svetlov P. G. (1924). Nablyudeniya nad Oligochaeta Permskoy gubernii. I. Materialy po faune, sistematike i ekologii dozhdevykh chervey [Observations over the Oligochaeta of the Perm province. Part I. Materials on the fauna, taxonomy, and ecology of earthworms]. Izvestiya Biologicheskogo Nauchno-Issledovatel’skogo Instituta pri Permskom Gosudarstvennom Universitete.

[B7467240] Vorobeichik E, Nesterkov A, Golovanova E, Nesterkova D, Ermakov A, Grebennikov M (2021). Long-term dynamics of the abundance of earthworms and enchytraeids (Annelida, Clitellata: Lumbricidae, Enchytraeidae) in forests of the Central Urals, Russia. Dataset/Samplingevent.. http://gbif.ru:8080/ipt/resource?r=lepc_annelids_1990-2020&v=1.1.

[B7467093] Vorobeichik E. L. (1998). Populations of earthworms (Lumbricidae) in forests of the Middle Urals in conditions of pollution by discharge from copper works. Russian Journal of Ecology.

[B7467111] Vsevolodova-Perel T. S. (1988). Rasprostraneniye dozhdevykh chervey na severe Palearktiki (v predelakh SSSR) [Distribution of earthworms in the north of the Palaearctic (within the USSR)]. Biologiya Pochv Severnoy Yevropy.

[B7467133] Vsevolodova-Perel T. S. (1997). Dozhdevyye chervi fauny Rossii: kadastr i opredelitel’.

[B7546276] WRB IUSS Working Group (2015). World Reference Base for Soil Resources 2014, update 2015. International soil classification system for naming soils and creating legends for soil maps..

